# Sleep disorders after cardiac arrest: Prevalence and relation with cognitive function

**DOI:** 10.1016/j.resplu.2025.100913

**Published:** 2025-02-21

**Authors:** A.B. Glimmerveen, J. Bos, E.G.J. Zandbergen, J. Hofmeijer, H.M. Keijzer

**Affiliations:** a Department of Neurology, Rijnstate Hospital, Arnhem, the Netherlands; b Clinical Neurophysiology, Technical Medical Centre, University of Twente, Enschede, the Netherlands

**Keywords:** Cardiac arrest survivors, Cognitive function, Sleep disorder

## Abstract

**Background:**

Long term cognitive impairment affects about half of cardiac arrest survivors, typically attributed to postanoxic encephalopathy. Sleep disorders are common after acute brain injuries and may also impair cognition. We investigated the prevalence of sleep disorders in cardiac arrest survivors and their relation with cognitive function.

**Method:**

Thirty survivors completed neuropsychological examination and questionnaires on mood (Hospital anxiety and depression scale), daytime sleepiness (Epworth sleepiness scale), and sleep quality (Pittsburgh sleep quality index), and underwent polysomnography one year after cardiac arrest. Questionnaire outcomes and objective sleep parameters (sleep-apnea, leg movements, cyclicity) were correlated with performance on neuropsychological tests using Pearson R, Kruskal-Wallis, or Mann-Whitney U tests.

**Results:**

Thirty-six percent of participants had moderate to severe obstructive sleep apnea, and 43% moderate to severe periodic limb movements during sleep according to polysomnography. Obstructive sleep apnea was correlated with poorer executive functioning (*R* = −0.38; *p* < 0.05) and memory (*R* = −0.50; *p* < 0.05). Fewer sleep cycles were correlated with poorer attention (*R* = 0.36, *p* = 0.05). Questionnaire outcomes (mood, daytime sleepiness, sleep quality) were not related to cognition. Participants with moderate/severe obstructive sleep apnea had worse executive functioning than those with no/mild obstructive sleep apnea (*p* = 0.02).

**Conclusion:**

This explorative study shows moderate to severe sleep disorders are common in cardiac arrest survivors and that moderate to severe obstructive sleep apnea relates to poorer cognitive function. This implies that diagnosis and treatment of obstructive sleep apnea may offer a treatment target for cardiac arrest survivors with cognitive impairment.

## Highlights

• Moderate to severe sleep disorders are common in cardiac arrest survivors.
• Moderate to severe sleep apnea relates to poorer cognitive function.
• Treatment of sleep disorders may be an additional therapy for cognitive disturbances after cardiac arrest.

## Introduction

Roughly half of cardiac arrest survivors faces persistent cognitive impairment, especially in the domains of memory, attention, and executive function.^1^, ^2^, ^3^, ^4^, ^5^ Direct postanoxic brain injury is probably the most important causal factor. However, disturbances of sleep may play a role.^6^

Sleep is a fundamental component of human health, playing a critical role in various cognitive processes.^7^, ^8^, ^9^ Adequate sleep is vital for the consolidation of declarative and procedural memory^7^, ^8^, ^10^ and sleep deprivation has been demonstrated to have significant neurocognitive consequences.^11^, ^12^ The impact of various sleep disorders on cognition is well-documented. Especially obstructive sleep apnea (OSA) has been associated with impairments of attention, executive function, and memory.^13^, ^14^, ^15^ There is also evidence of a relation between periodic limb movement disorder (PLMD) and poor cognitive functioning.^16^, ^17^

Several neurological conditions are known to affect sleep and can induce sleep disorders such as OSA and PLMD. Stroke, for example, has a well-established association of insomnia (prevalence in stroke 27–38%),^18^, ^19^ sleep-disordered breathing (50–72%),^20^, ^21^, ^22^, ^23^ and restless leg syndrome (2–12%).^24^, ^25^ Also heart failure, depression, cerebral small vessel disease, Alzheimer’s disease and Parkinson's disease are characterized by increased rates of sleep disorders. In turn, sleep disorders are associated with poorer long term outcomes in these populations.^26^, ^27^, ^28^, ^29^, ^30^, ^31^, ^32^ This highlights the complex interplay between brain diseases, systemic conditions, and sleep health.^33^

We hypothesize that sleep disorders are relatively prevalent amongst cardiac arrest survivors and associated with relatively poor cognitive functioning. To test these hypotheses, we investigated the long term prevalence and phenotypes of sleep disturbances in a prospectively collected cohort of survivors after cardiac arrest, including the relationship between the presence of a sleep disturbance and cognitive functioning. Our results may provide a starting point for additional treatment options for patients with long term cognitive impairment after cardiac arrest.

## Methods

The Brain Outcome after Cardiac Arrest (BROCA)-prediction study (NL9451, Dutch Trial Register) is a prospective longitudinal multicenter cohort study, designed to develop a multimodal prediction model for long-term participation in society and cognitive outcome of survivors after cardiac arrest. Inclusion in BROCA started in November 2019 and is ongoing in six Dutch hospitals. We collect clinical, EEG, and brain-MRI data during hospital admission and follow patients for one year. In general, recovery after cardiac arrest reaches a steady state after twelve months.^34^ Follow up includes neuropsychological examination at twelve months after cardiac arrest. In one hospital (Rijnstate Hospital), we also collect polysomnography data at twelve months during the period of November 1st, 2019, and November 30th, 2023. The medical Ethical Committee Arnhem/Nijmegen approved the protocol (NL69767.091.19).

### Patients and in-/exclusion criteria

We included awake patients after successful resuscitation on cardiology departments in the BROCA study. This indicates that we included patients that were discharged from the intensive care unit (ICU) after recovery of consciousness, and patients that were directly admitted to cardiology departments. We excluded patients who were resuscitated in the ambulance on their way to the hospital for another indication than cardiac arrest. Other exclusion criteria were preexistent brain damage with a modified Rankin Scale score > 2, life expectancy of less than three months because of another medical condition, and insufficient knowledge of Dutch language to fill out questionnaires.

For the current explorative analysis, we used data from thirty patients with polysomnography and neuropsychological examination (NPE) collected twelve months after cardiac arrest.

### Cognitive outcome measures

Patients were evaluated with neuropsychological tests at twelve months following cardiac arrest, focusing on memory (Rey Auditory Verbal Learning Test (RAVLT)), attention (Trail Making Test A & B (TMT-AB) and Stroop Color and Word Test), and executive functioning (TMT-AB, Stroop, short Raven’s progressive matrices, and letter fluency). Z-scores for RAVLT, TMT-AB, and Stroop were computed using Maasnorms, adjusted for age, sex and educational level.^35^ Norms for the Short Raven were based on previous research,^36^ while letter fluency norms adhered to the Netherlands Institute for Psychologists (NIP) guidelines.^37^ For every patient, a composite z-score for each cognitive domain was derived by averaging the individual z-scores of the subtests within each domain (supplementary material S1). A patient was considered impaired in a cognitive domain when this composite z-score was ≥ 1.5 SD below the mean.

### Polysomnography recordings

Home-based ambulant polysomnography registrations were recorded at twelve months after cardiac arrest using Morpheus recorders (Micromed, Italy). Registrations were in line with the American Association of Sleep Medicine (AASM) guidelines for ambulant polysomnography recordings. Accordingly, the sleep brain pattern was measured with EEG electrodes on the frontal (F3, F4), central (C3, C4), and occipital areas (O1, O2) and eye movements with one electrode just above the right eye (lateral side) and one electrode just below the left eye (lateral side). Muscle tone was measured with EMG electrodes on the chin and bilaterally on the m. tibial anterior. Ventilation was measured with thoracic and abdominal straps, a nasal cannula, and a thermistor. A piezoelectric sensor measured levels of snoring. Blood oxygen levels were measured with a flexible saturation sensor attached to a finger of the non-dominant hand. Body position was measured with a sensor on the recorder.

### Polysomnography parameters and analysis

One somnotechnician (JB) scored all polysomnographic recordings with Brain-RT software (OSG/NATUS, Belgium). The following parameters were calculated for analysis: number of minutes of the different sleep stages (Non-rapid eye movement (NREM) I, NREM II, NREM III, REM), number of minutes until start of sleep (sleep latency), sleep efficiency, apnea/hypopnea index (AHI; based on the 3% desaturation rule), and periodic limb movement during sleep index (PLMS). Normative values for the different sleep stages and sleep efficiency were based on the AASM criteria and summarized in supplementary material S2A. The classification of PLMS and OSA was based on the parameters PLMS and AHI, respectively, and according to the AASM criteria. Depending on the parameter value, patients were categorized as having no, mild, moderate or severe PLMS or OSA (see supplementary material S2B). Evaluation of the hypnogram (cyclicity) was based on visual analysis by the somnotechnician.

### Questionnaires

Patients were asked to fill out the Hospital anxiety and depressions scale (HADS) and two sleep related questionnaires: the Pittsburgh sleep quality index (PSQI) and the Epworth sleepiness scale (ESS).

The HADS is a self-report scale and consists of 14 items to measure symptoms of anxiety (HADS-A) or depression (HADS-D).^38^ Scores range between 0 and 21 per subscale and may provide evidence for mild symptoms (score ≥ 8) or severe symptoms (score ≥ 11) of depression or anxiety.

The PSQI is a patient reported questionnaire to assess sleep quality over a one month interval.^39^ The questionnaire consists of nine questions reflecting the patient’s experience of sleep and one question reflecting the bedpartner’s or roommate’s experience of the patient’s sleep behavior. Seven components of sleep are derived from the questionnaire: 1) subjective sleep quality, 2) sleep latency, 3) sleep duration, 4) sleep efficiency, 5) sleep disturbance, 6) use of sleep medication, and 7) daytime dysfunction. The global PSQI score is the sum of the seven component scores and ranges from 0 to 21. A global PSQI score of 5 or higher indicates a “poor” sleeper.

The ESS is designed to measure the general level of daytime sleepiness.^40^ Patients are asked to rate how likely they are to doze off in 8 situations, with a score ranging from 0 (never) to 3 (high chance). An ESS score of 10 or higher indicates a relatively high level of daytime sleepiness.

### Statistical analysis

Demographic information is presented as median and interquartile range (IQR) for continuous variables, and number and percentage for categorical variables.

We present long term cognitive functioning as median normalized cognitive scores per test and per domain, as well as the percentage of impaired patients (i.e., patients with a z-score ≥ 1,5 SD below the normative mean).

We present continuous variables as median [IQR] and categorical variables as numbers and percentage. To indicate the relation between sleep score and long term cognitive outcome, we used Pearson R correlation tests correlating ESS, PSQI, HADS-A, HADS-D, AHI, PLMS, and cyclicity to the three cognitive domains. In addition, we calculated Pearson’s R between the questionnaires (PSQI, ESS, HADS-A, and HADS-D) and the polysomnography parameters (AHI, PLMS, and cyclicity). Finally, we tested whether the questionnaires (PSQI, ESS, HADS-A, and HADS-D) related with each other.

Based on the parameters AHI and PLMS, patients are classified as no, mild, moderate, or severe OSA or PLMS. We used ANOVA or Kruskal Willis or Mann Whitney *U* test, where appropriate, to analyze between-group differences in cognitive performance between the OSA and PLMS groups. We performed a post hoc analysis where we dichotomized these groups as no/mild OSA, PLMS or cyclicity, versus moderate/severe OSA, PLMS or cyclicity. We did not correct for multiple comparisons because of the explorative nature of these analyses.

All statistical analyses are performed with R (version R.4.3.1; https://www.R-project.org) and Rstudio.

## Results

**Fig. 1:**
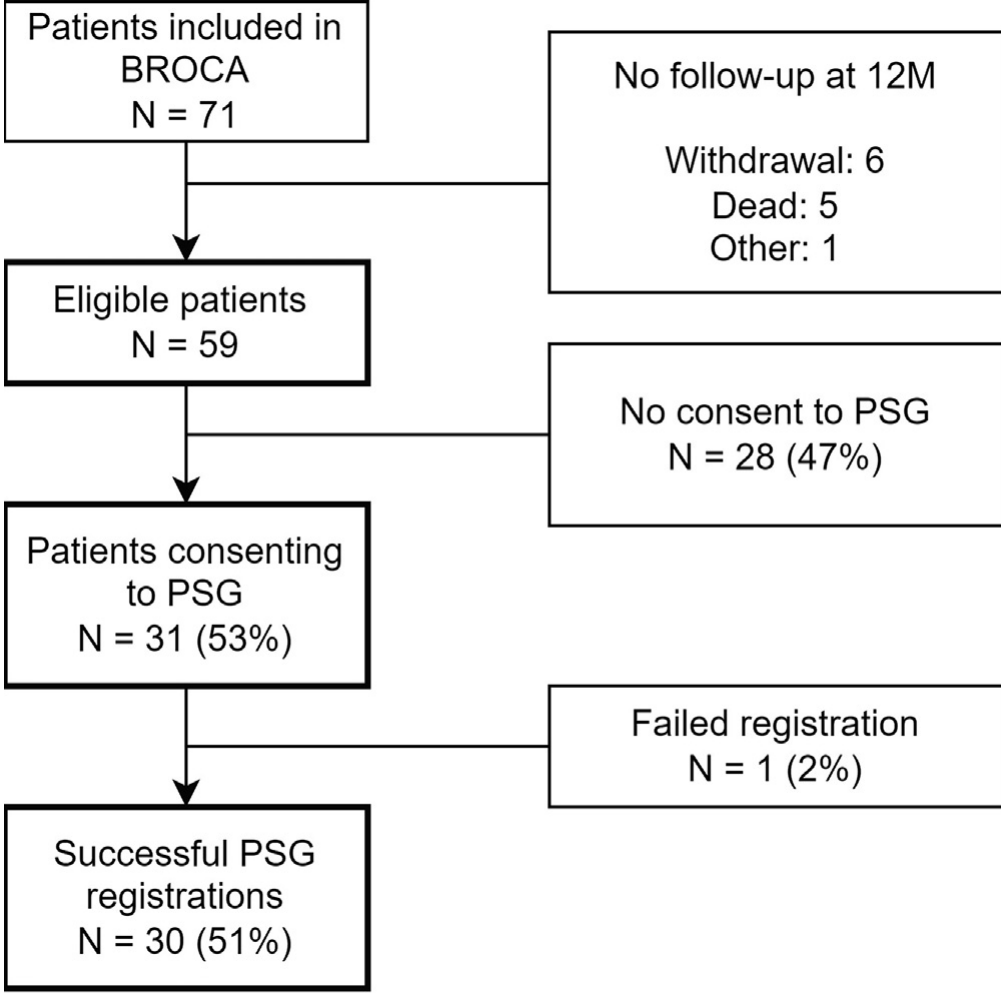
Flow of patients through the BROCA prediction study at Rijnstate Hospital Arnhem, in the period of November 1st, 2019, and November 30th, 2023.

We included 71 cardiac arrest survivors in the BROCA study in Rijnstate hospital, of whom 31 consented to polysomnography at twelve months after cardiac arrest. One registration failed, leaving thirty patients for this analysis (Fig. 1).

### Baseline characteristics

Baseline characteristics of our sample are presented in Table 1. The majority of our patients was male (87%) and the median age was 65.5 years. Baseline characteristics of patients included in our sleep analysis were essentially similar as those that refused polysomnography measurements, except for level of anxiety, which was slightly higher in those refusing (2 vs. 4, P < 0.05).

**Table 1:** Patient characteristics and mood and cognition scores at twelve months.

	Subjects with successful PSG and cognitive evaluation (n = 30)		Subjects without PSG (n = 41)	*p*
**Hospitalization**				
Sex (male)	26 (87%)		33 (80%)	0.54
Age (years)	65.5 [59.3–68]		64 [53–73.3]	0.08
BMI	28.1 [24.2–28.9]		26.8 [25.1–29.6]	0.50
ROSC (days)	10 [9.8–15]		13.5 [10–19.5]	0.30
Cause of arrest				0.52
Acute coronary syndrome	19 (63%)		29 (71%)	
Cardiomyopathy	2 (7%)		1 (2%)	
Monogenic arrhythmias	0 (0%)		1 (2%)	
Other	5 (17%)		5 (12%)	
Unknown	4 (13%)		5 (12%)	
Initial rhythm				0.58
Shockable	24 (80%)		30 (73%)	
Non-shockable	0 (0%)		0 (0%)	
Unknown	6 (20%)		11 (27%)	
Shock administered by				0.65
AED	8 (27%)		8 (20%)	
Ambulance	10 (33%)		12 (29%)	
Both	11 (37%)		19 (46%)	
Unknown	1 (3%)		2 (5%)	
ICU stay (yes)	22 (73%)		26 (63%)	0.45
Days of ventilation	1 [1–2]		1 [1–2.3]	0.42
Dose midazolam (mg/kg/h)	0.058 [0.038–0.068]		0.038 [0.034–0.046]	0.36
Dose morphine (mg/kg/h)	0.017 [0.015–0.025]		0.021 [0.017–0.024]	0.60
Dose propofol (mg/kg/h)	2.740 [2.106–3.839]		2.348 [2.035–3.188]	0.71
ICU + CCU (days)	11 [7.5–19.5]		8 [5–18]	0.36
Delirium (yes)	7 (24%)		13 (32%)	0.59
Discharge				0.07
Home	26 (87%)		27 (66%)	
Rehabilitation	3 (10%)		4 (10%)	
Other	0		6 (15%)	
Unknown	1 (3%)		4 (10%)	
**12 months follow-up**				
Mood				
HADS anxiety score	2 [1–4]		4 [1–7]	
HADS depression score	2 [1–5.3]		3 [1–7]	
Cognition	Score	Impaired (%)		
Attention domain	−0.41 [−1.05–0.35]	13%		
Executive functioning domain	−0.33 [−0.92–0.11]	17%		
Memory domain	−0.92 [−1.40–−0.32]	17%		

Demographic variables are presented as median [IQR] for continuous variables and number (%) for categorical variables. Mood and cognitive variables are presented as median [IQR] and % of patient with scores ≥ 1.5 SD below the mean for subjects with (N = 30).

PSG = polysomnography; BMI = Body Mass Index; ROSC = Return of spontaneous circulation; AED = automated external defibrillator; ICU = Intensive Care Unit; CCU = Cardiac Care Unit; HADS = Hospital Anxiety and Depression Scale.

### Cognitive outcome

Median z-scores were below zero (i.e., lower than the average of the norm population) in all three cognitive domains. Thirteen percent scored on impairment level (≥1.5 SD below the normative mean) for attention, 17% for executive functioning, and 17% for memory. Data are summarized in Table 1. The individual cognitive test scores can be found in supplementary material S3.

### Sleep parameters

Three patients reported increased levels of daytime sleepiness based on the ESS, and eighteen patients reported poor sleep quality based on the PSQI. Polysomnographic measurements showed that median amounts of light sleep (NREM I + II), deep sleep (NREM III), REM sleep, number of sleep cycles, and sleep efficiency were within the norm. Moderate to severe OSA was found in eleven (36%) of the patients, and moderate to severe PLMS in thirteen (43%). All sleep parameters are summarized in Table 2.

**Table 2:** Summary of scores on the sleep questionnaires, sleep parameters, and potential sleep disturbers.

	Patients (n = 30)	Normal values
**Questionnaires**		
PSQI score	5 [4–7.8]	<5
ESS score	5 [1.3–7]	<10
**Polysomnography results**		
**Sleep**		*
NREM I + II (%)	64.6 [57.9–71.0]	55–62%*
NREM III (%)	17.9 [14.1–22.4]	10–15%*
REM (%)	20 [14.1–24.1]	15–20%*
Sleep latency (min)	7.25 [3.7–12.4]	<30 min
Sleep efficiency (%)	85.7 [81.3–90.5]	80–95%*
Cycles (#)	4 [4–5]	
**Potential sleep disturbers**		
*Respiratory*		
AHI (/h)	7.4 [4.7–27.2]	
*Movement*		
PLMS (/h)	24 [3.6–40.8]	
**Classification of sleep disturbers**		
*Respiratory*		
No OSA (AHI <5 per hour)	10 (33%)	
Mild OSA (AHI ≥5, but <15 per hour)	9 (30%)	
Moderate OSA (AHI ≥15, but <30 per hour)	4 (13%)	
Severe OSA (AHI ≥30 per hour)	7 (23%)	
*Movement*		
No PLMS (index <5 per hour)	9 (30%)	
Mild PLMS (index ≥5, but <25 per hour)	8 (27%)	
Moderate PLMS (index ≥25, but <50 per hour)	7 (23%)	
Severe PLMS (index ≥50 per hour)	6 (20%)	

Data are presented as median [IQR] or number (%). PSQI = Pittsburgh Sleep Quality Index; ESS = Epworth Sleepiness Scale; NREM = non-rapid eye movement; AHI = apnea/hypopnea index; AI = apnea index; OSA = obstructive sleep apnea, PLMS = periodic limb movement in sleep. *= distribution of sleep within the average population.

### Relation between sleep parameters and cognitive outcomes

From polysomnography, a higher AHI was correlated with poorer scores in the cognitive domains of executive functioning (Pearson *R* = −0.38; *p* < 0.05) and memory (Pearson *R* = −0.50; *p* < 0.05). A similar (but non-significant) relation was observed for attention (Pearson *R* = −0.35; *p* = 0.06). A reduced number of sleep cycles was correlated with poorer performance in the attention domain (Pearson *R =* 0.36, *P* = 0.05), but not with performance in the other domains. PLMS was not correlated with performance in any of the cognitive domains.

From the questionnaires, signs of depression (HADS-D), anxiety (HADS-A), daytime sleepiness (ESS), or sleep quality (PSQI) did not show any significant correlations with performance in any of the cognitive domains (supplementary material S4). Better sleep quality was related to less signs of anxiety (Pearson *R* = 0.53, *p* < 0.05), but not to signs of depression, daytime sleepiness, or the polysomnography parameters AHI, PLMS index, or cyclicity.

**Fig. 2:**
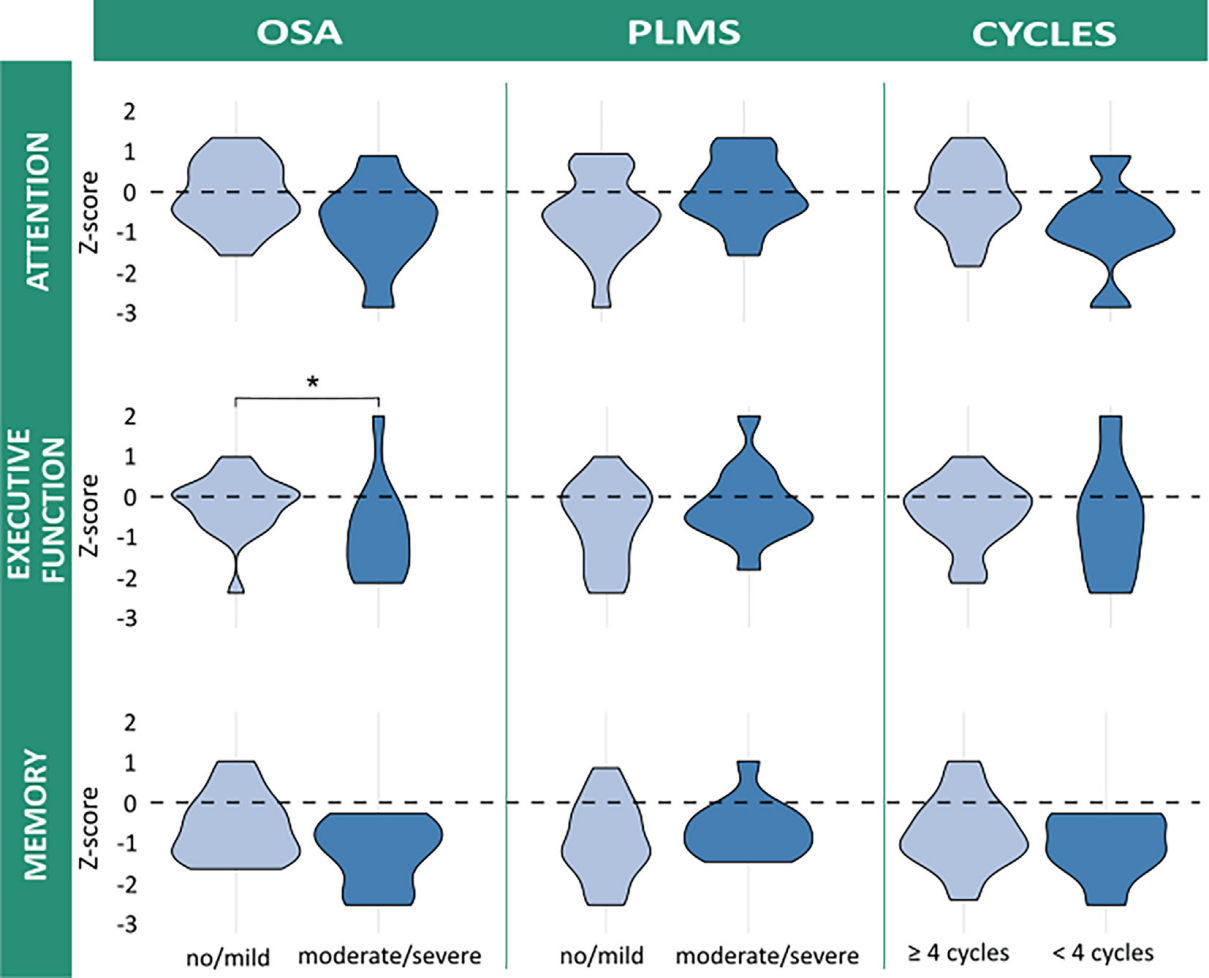
Representation of relation between cognitive performance (z-scores) and OSA, PLMS, and cyclicity of sleep. The dashed line indicates a z-score of 0. Significant between-group differences (p < 0.05) are represented with *.

We found no significant differences in performance on the three cognitive domains between groups of patients with no, mild, moderate, and severe OSA, or the different levels of PLMS. However, after dichotomizing, the group with moderate/severe OSA performed worse in the domain of executive functioning than the no/mild OSA group (W_exe_ = 160.0, *p* = 0.02). A similar (but non-significant) relation was observed in the attention and memory domains (W_att_ = 150.0, *p* = 0.05; W_mem_ = 148.5, *p* = 0.06) (Fig. 2).

## Discussion

In this prospective cohort of thirty survivors after cardiac arrest, we found that 36% of participants had moderate to severe OSA and 43% moderate to severe PLMS at one year after cardiac arrest. We observed a negative relation between OSA severity and cognitive performance, particularly in the executive functioning and memory domains. No significant relations were found between PLMS, cyclicity of sleep, or other PSG derived parameters and functioning in the various cognitive domains.

To our knowledge, no previous study investigated the relationship between subjective or objective sleep parameters and long term cognitive functioning in cardiac arrest survivors. However, our observed relation between OSA and cognitive functioning and impairment aligns with existing literature indicating that untreated OSA can lead to cognitive decline.^41^ Cognitive impairment associated with OSA is typically manifest in the domains of executive functioning, attention and memory. This is similar to cognitive impairment observed in cardiac arrest survivors. Importantly, OSA is a treatable condition, unlike postanoxic encephalopathy. Effective treatment of OSA, such as with continuous positive airway pressure (CPAP) therapy, has been shown to improve global cognitive functioning.^42^, ^43^ Although OSA treatment will probably have little impact on postanoxic encephalopathy-induced impairments, it may still provide a treatment target to improve cognitive functioning. This underlines the relevance of considering OSA in the management of cardiac arrest survivors with cognitive impairment.

OSA and PLMD are common sleep disorders. In Europe, about 8–21% suffer from moderate to severe OSA^44^, ^45^ and 5–8% of adults experience PLMD.^46^ Our study revealed a higher prevalence of these conditions in survivors of cardiac arrest than in the general population. This is in line with studies after stroke, where even higher prevalences of OSA have been reported.^20^, ^21^, ^22^, ^23^ The relatively high prevalence of sleep disorders in our cohort and in other patients with acute brain injury could potentially be explained by emotional disturbances such as anxiety and depression. These are also common after cardiac arrest.^47^ However, in our current study population, none of the patients exhibited significant signs of anxiety, and only two had mild signs of depression. In addition, our observed prevalence of OSA is higher than the reported 10–11% for overweight people with a comparable BMI to our population (25 ≤ BMI < 30 kg/m^2^).^48^ This all suggests that other factors may be at play. We speculate that, as in neurodegenerative diseases,^28^, ^31^ hypoxic-ischemic brain injury may cause sleep disturbances in itself.

Despite sixty percent of our patients reporting poor sleep quality (as measured by the PSQI), they did not report experiencing daytime sleepiness (as measured by the ESS). In addition, results on PSQI and ESS questionnaires were not related to the polysomnography parameters. This underscores the possible discrepancy between objective measures and subjective complaints.^49^, ^50^ We did find a relationship between PSQI scores and signs of anxiety, which supports previous research suggesting that PSQI is more closely related to psychological symptoms than to daytime sleepiness.^49^

Prior studies reported inconsistent results concerning the association between PLMS and cognition, with some demonstrating a clear relation^16^ and other failing to do so.^17^ Opposite to OSA, we did not find a relationship between PLMS and cognitive functioning or impairment. However, in patients with other neurological conditions such as cerebral small vessel disease or Alzheimer's disease, the presence of PLMD can exacerbate cognitive deficits.^30^, ^31^ Therefore, monitoring the periodic limb movements in sleep in cardiac arrest survivors who present with cognitive complaints may still have clinical relevance.

### Strengths and limitations

Strengths of our study include the prospective design, the use of validated sleep and cognitive testing instruments, and state of the art measurements and scorings, blinded to cognitive functioning of the patients.

There are also several limitations. First, our sample was relatively small (*N* = 30), hampering statistical power. Second, there was a potential selection bias, since patients who were more severely affected were less likely to participate in one year cognitive testing and polysomnography. This may explain the relatively low prevalence of cognitive impairment in cardiac arrest survivors compared to the whole sample of patients included in the BROCA study,^51^ as well as lower prevalence of sleep disturbances found in our population compared to patients after ischemic stroke. Finally, we do not have polysomnography data from before the cardiac arrest, which limits our ability to conclusively attribute the onset of sleep disorders to the cardiac event. However, many patients informed the technicians that their sleep quality had significantly declined since the arrest.

## Conclusion

This explorative study shows that moderate to severe OSA is common in cardiac arrest survivors and associates to poorer cognitive functioning. This may imply that diagnosis and treatment of OSA may offer an additional treatment target for cardiac arrest survivors with cognitive impairment.

## Ethical statement

The medical Ethical Committee Arnhem/Nijmegen approved the protocol (NL69767.091.19) on 27-05-2019.

## Funding source

Jeannette Hofmeijer is supported by a clinical established investigator grant of the Dutch Heart Foundation (Grant Number 2018T070).

## CRediT authorship contribution statement

**A.B. Glimmerveen:** Conceptualization, Formal analysis, Investigation, Methodology, Software, Visualization, Writing – original draft. **J. Bos:** Formal analysis, Investigation, Methodology, Software, Writing – review & editing. **E.G.J. Zandbergen:** Formal analysis, Methodology, Writing – review & editing. **J. Hofmeijer:** Conceptualization, Formal analysis, Methodology, Supervision, Writing – review & editing. **H.M. Keijzer:** Conceptualization, Formal analysis, Methodology, Software, Supervision, Writing – review & editing.

## Declaration of competing interest

The authors declare that they have no known competing financial interests or personal relationships that could have appeared to influence the work reported in this paper.
