# Supplementary material for: Sleep disorders after cardiac arrest: Prevalence and relation with cognitive function
Source: Resusc Plus. 2025 Feb 21;22:100913. doi: 10.1016/j.resplu.2025.100913 (PMC11929073; doi:10.1016/j.resplu.2025.100913)
Supplement: Supplementary Data 1 [file mmc1.docx]

**Supplementary material**

S1. Overview of the neuropsychological tests per cognitive domain. Composite Z-scores are computed per cognitive domain by dividing the sum of the individual z-score per subtest by the number of subtests.

| Domain | Test |
| --- | --- |
| Attention | TMT-A (time)  Stroop-I (time)  Stroop-II (time)  Stroop-III (time) |
| Executive functioning | Short Raven  TMT B corrected for A  Stroop inference  Letter fluency (KOM) |
| Memory | RAVLT total recall trial 1-5  RAVLT delayed recall  RAVLT recognition |

TMT = Trail-Making-Test; RAVL = Rey Auditory Verbal Learning Test
