# Supplementary material for: Sleep disorders after cardiac arrest: Prevalence and relation with cognitive function
Source: Resusc Plus. 2025 Feb 21;22:100913. doi: 10.1016/j.resplu.2025.100913 (PMC11929073; doi:10.1016/j.resplu.2025.100913)
Supplement: Supplementary Data 2 [file mmc2.docx]

**Supplementary material**

S2.A & S2.B. Normative values used for the evaluation of the distribution of sleep stages, sleep efficiency and the classification of PLMS and OSA. All based on AASM data.

S2.A

Distribution of sleep stages and sleep efficiency

| Age: | > 50 jaar |
| --- | --- |
|  |  |
| NREM I & II | 0-62% |
| NREM II | 50-55% |
| NREM III | 10-15% |
| REM | 15-20% |
|  |  |
| Sleep efficiency | 80-95% |

(N)REM = (non )-rapid eye movement

S2.B

Classification of PLMS and OSA

|  | PLMS index |  | AHI |
| --- | --- | --- | --- |
| Mild PLMS: | ≥5, but <25 per hour | Mild OSA: | ≥5, but <15 per hour |
| Moderate PLMS: | ≥25, but <50 per hour | Moderate OSA: | ≥15, but < 30 per hour |
| Severe PLMS: | ≥ 50 or PLMS-arousal-index ≥ 25 per hour | Severe OSA: | ≥30 per hour |

PLMS = Periodic limb movement in sleep; OSA = Obstructive sleep apnea; AHI = apnea/hypopnea index
