# Supplementary material for: Sleep disorders after cardiac arrest: Prevalence and relation with cognitive function
Source: Resusc Plus. 2025 Feb 21;22:100913. doi: 10.1016/j.resplu.2025.100913 (PMC11929073; doi:10.1016/j.resplu.2025.100913)
Supplement: Supplementary Data 3 [file mmc3.docx]

**Supplementary material**

S3. Scores for cognitive domains and individual tests are presented as median [IQR] and % of scores ≤ 1.5 SD below the normative mean for subjects.

| Cognition  *Attention domain*  TMT-A  Stroop-I  Stroop-II  Stroop-III | -0.41 [-1.05 – 0.35]  0.13 [-0.70 – 0.89]  -0.71 [-1.72 – 0.27]  -0.44 [-1.03 – 0.35]  -0.09 [-0.70 – 0.71] | 13%  10%  33%  17%  13% |
| --- | --- | --- |
|  |  |  |
| *Executive functioning domain*  Short Raven  TMT B corrected for A  Stroop inference  Letter fluency (KOM) | -0.33 [-0.92 – 0.11]  -1.33 [-1.75 – 0.19]  -0.33 [-1.08 – 0.10]  0.15 [-0.49 – 0.73]  -0.85 [-1.42 – -0.30] | 17%  27%  17%  13%  25% |
|  |  |  |
| *Memory domain*  RAVLT total recall  RAVLT delayed recall  RAVLT recognition | -0.92 [-1.40 – -0.32]  -1.11 [-1.88 – -0.48]  -1.37 [-1.88 – -0.82]  -0.20 [-0.63 – 0.32] | 17%  37%  47%  10% |

TMT = Trail-Making-Test; RAVL = Rey Auditory Verbal Learning Test
