# Supplementary material for: Sleep disorders after cardiac arrest: Prevalence and relation with cognitive function
Source: Resusc Plus. 2025 Feb 21;22:100913. doi: 10.1016/j.resplu.2025.100913 (PMC11929073; doi:10.1016/j.resplu.2025.100913)
Supplement: Supplementary Data 4 [file mmc4.docx]

**Supplementary material**

S4. Correlation matrix with Pearson R coefficients for correlations between different parameters and cognitive domains.

|  | Attention | Executive  Functioning | Memory |
| --- | --- | --- | --- |
| Anxiety (HADS) | 0.09  *P* = 0.65 | -0.08  *P* = 0.70 | -0.12  *P* = 0.56 |
| Depression (HADS) | -0.26  *P* = 0.18 | -0.12  *P* = 0.56 | -0.23  *P* = 0.24 |
| ESS score | 0.23  *P* = 0.23 | 0.14  *P* = 0.46 | 0.30  *P* = 0.11 |
| PSQI score | -0.12  *P* = 0.54 | -0.21  *P* = 0.27 | 0.04  *P* = 0.85 |
| AHI | -0.35  *P* = 0.06 | -0.38  *P* = **0.04** | -0.50  *P* **< 0.01** |
| PLMS index | 0.25  *P* = 0.18 | 0.22  *P* = 0.24 | 0.09  *P* = 0.65 |
| Cyclicity | 0.36  *P* = **0.05** | 0.19  *P* = 0.30 | 0.15  *P* = 0.39 |

HADS = Hospital anxiety and depression scale; ESS = Epworth sleepiness scale; PSQI = Pittsburgh sleep quality index; PLMS = Periodic limb movement in sleep; OSA = Obstructive sleep apnea; AHI = apnea/hypopnea index
